# Spatially and Spectrally Concatenated Neural Networks for Efficient Lossless Compression of Hyperspectral Imagery

**DOI:** 10.3390/jimaging6060038

**Published:** 2020-05-28

**Authors:** Zhuocheng Jiang, W. David Pan, Hongda Shen

**Affiliations:** 1 Department of Electrical and Computer Engineering, University of Alabama in Huntsville, Huntsville, AL 35899, USA; zj0004@uah.edu; 2 Chubbs Insurance Inc., New York, NY 10020, USA; hs0017@alumni.uah.edu

**Keywords:** predictive lossless compression, hyperspectral imagery, neural networks, onboard data compression, spatial and spectral correlations

## Abstract

To achieve efficient lossless compression of hyperspectral images, we design a concatenated neural network, which is capable of extracting both spatial and spectral correlations for accurate pixel value prediction. Unlike conventional neural network based methods in the literature, the proposed neural network functions as an adaptive filter, thereby eliminating the need for pre-training using decompressed data. To meet the demand for low-complexity onboard processing, we use a shallow network with only two hidden layers for efficient feature extraction and predictive filtering. Extensive simulations on commonly used hyperspectral datasets and the standard CCSDS test datasets show that the proposed approach attains significant improvements over several other state-of-the-art methods, including standard compressors such as ESA, CCSDS-122, and CCSDS-123.

## 1. Introduction

Hyperspectral sensors collect data as a set of images with high spatial and spectral resolutions, with each spectral band image being a narrow wavelength range of the electromagnetic spectrum. The large quantities of hyperspectral data present great challenges in storage, transmission, and analysis, as a consequence, data compression is becoming a common process for such imagery. In general, compression can be either lossy or lossless. Lossy compression typically provides lower bit rates but incurs loss on the original data. On the other hand, lossless compression guarantees perfect reconstruction on the original data, albeit with higher bit rates. This work focuses on improving the performance of onboard predictive lossless compression on hyperspectral imagery. The techniques are useful for many precision-demanding applications where strictly no data loss is highly desirable [1,2,3]. Below is a brief survey on the existing work on the subject.

Lossless compression of hyperspectral images has been performed very successfully using prediction-based methods. Context-based Adaptive Lossless Image Codec (3D-CALIC) [4] and its variant M-CALIC [5] consider both interband and intraband correlations to reduce prediction errors. The Lookup Table (LUT) approach in [6] exploits the calibration-induced data correlation specific to hyperspectral imagery to facilitate accuracy prediction. This scheme was enhanced by a Locally Averaged Interband Scaling (LAIS-LUT) approach using a band adaptive quantization factor [7].

Transform-based approaches such as Discrete Wavelet Transform (DWT) and Principal Component Analysis (PCA), aim to exploit the relations in the spectral and spatial dimensions based on a redundancy reduction transform. The problem of selecting an appropriate signal representation for transform-based compression is equivalent to the feature extraction problem in machine learning. Recently, Wavelet-based Regression Analysis (RWA) [8,9] was introduced for lossless compression by exploiting the relationships among wavelet-transformed components, which outperforms the traditional approaches.

Low-complexity filter-based compressors, such as the Fast Lossless (FL) [10] and Spectral-Oriented Least Squares (SLSQ) [11], utilize linear models to de-correlate the co-located pixels from different spectral bands. An optimized version of the Fast Lossless (FL) algorithm developed by the NASA Jet Propulsion Laboratory (JPL) has been selected as the core predictor in the new Consultative Committee for Space Data Systems (CCSDS) standard for multispectral and hyperspectral data compression [12]. Besides, traditional Wiener filter, Kalman filter and least mean square filter, were adopted for hyperspectral image compression. Examples include the Backward Pixel Search (BPS) [13], Kalman Spectral Prediction (KSP) [14] and Maximum Correntropy Criterion based Least Mean Square (MCC-LMS) [15] algorithms. Similar to linear predictors, nonlinear predictors such as Context-based Condition Average Prediction (CCAP) [16] and Two-Stage Prediction (TSP) [17] have also brought improvement in compressed bit rates.

High-complexity compressors such as Clustered Differential Pulse Code Modulation (C-DPCM) have been studied in [18], which partitions the data into several clusters with similar statistics and applies separate least-square optimized linear predictors to different clusters. [19] presents an Adaptive Prediction Length C-DPCM (C-DPCM-APL) method, which is a brute-force variant of the C-DPCM approach, in that the number of previous bands selected for prediction was determined by a brute-force search ranging from 10 to 200 bands in steps of 10. Two other C-DPCM variants also use a large portion of previous bands for prediction, including the Spectral Relaxation Labeled Prediction (S-RLP) and Spectral Fuzzy Matching Pursuits (S-FMP) in [20]. However, the computational complexity of these clustering-based compressors is very high.

Recently, Deep-learning based approaches have been widely utilized to lossy and lossless hyperspectral data compression. For lossy compression, [21,22,23,24] focused on designing deep networks to reconstruct the original imagery with a reasonable loss of information. Those models have an encoder-decoder structure, where representative features are usually extracted by Autoencoder network (AE) or Convolution Neural Network (CNN). Ref. [25] proposed an onboard CNN-based lossy compressor, where the neural network is pre-trained on other datasets in a ground-based setting. For lossless compression, deep neural networks [26,27] and recurrent neural networks (RNN) [28] have been proposed to compress hyperspectral data by appropriately pre-training the networks.

Nonetheless, the above mentioned deep-learning methods are not suitable for the challenging task of onboard lossless compression of hyperspectral images. The main reason is that deep learning relies on the availability of data from all the spectral bands during the process of training or clustering. However, either the entire original dataset or decompressed dataset is normally not available, or only partially available in many real-time compression applications. Furthermore, a pre-trained model can not in general adapt well for some new datasets, which necessitates model retraining for each new dataset.

To address those limitations, we propose an adaptive filtering-based Concatenated Shallow Neural Network (CSNN) model for predictive lossless compression. The contributions of the proposed method are twofold: (1) The CSNN was designed as an adaptive prediction filter rather than as a training-based network. Thus the model needs not be pre-trained before being used for pixel value calculation. To the best of our knowledge, this might be the first neural network based method requiring no training proposed for hyperspectral data compression. (2) The shallow two-hidden layer structure of the proposed model is capable of capturing both spatial and spectral correlations to provide more accurate pixel prediction, with only a few contexts from four previous bands. Consequently, computational complexity is much lower than other deep-learning based methods.

The rest of the paper is organized as follows. Section 2 discusses context selection for prediction and provides an information theoretic analysis of the prediction performance. Section 3 describes the proposed method in detail. Simulation results are given in Section 4. Finally, conclusions are drawn in Section 5.

## 2. Context Selection and Prediction Performance Analysis

Let $s_{x,y,z}$ denotes the pixel value at line *x* and column *y* in band *z* of a hyperspectral image cube. Rather than directly encoding the value of $s_{x,y,z}$, a predictive compression algorithm uses previously decoded pixel values to compute a predicted pixel value ${\hat{s}}_{x,y,z}$. Then the prediction residual, ($s_{x,y,z}-{\hat{s}}_{x,y,z}$), which is the difference between the actual pixel value and its estimate is encoded losslessly using an entropy coder.

### 2.1. Context Selection

The predictor attempts to exploit correlations between the contexts and the current pixel value. Thus, the first step is to select the contexts appropriately. Typically, the neighboring pixels tend to have correlations. Considering the fact that spectral correlations tend to be much stronger than spatial correlations, the FL and MCC-LMS methods use only spectral contexts, while CCAP, TSP and M-CALIC methods combine spatial context with spectral context for prediction. Following the practice in [10], as a pre-processing step, we perform a simple local averaging to better exploit the spatial correlations as follows:

(1) $${\bar{s}}_{x,y,z}=\frac{1}{4}\left(s_{x-1,y,z}+s_{x-1,y-1,z}+s_{x,y-1,z}+s_{x-1,y+1,z}\right)$$

**Figure 1:**
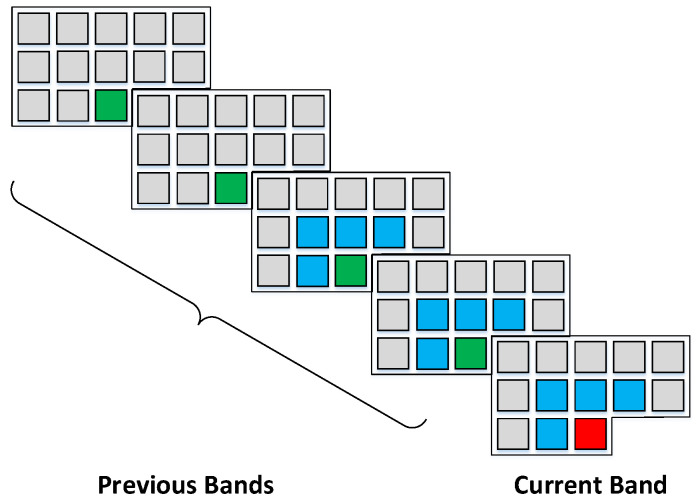
An example of context selection scheme for predicting the current pixel (in red), which consists of spatially neighboring pixels from the current band and two previous bands (in blue), as well as spectrally co-located pixels from the four previous bands (in green).

Figure 1 shows an example of context selection. The spatial context was selected from the current band and two previous bands. For each band, four neighboring pixels are reshaped into a 1-D vector $\left\{{\bar{s}}_{x,y-1,z},{\bar{s}}_{x-1,y-1,z},{\bar{s}}_{x-1,y,z},{\bar{s}}_{x-1,y+1,z}\right\}$, note the selected pixels are averaged values. Thus, the combined spatial context, denotes as $C_{t}$, is a 3 × 4 matrix. If a pixel reaches the boundary of image, we can still use the four nearest pixels to construct the context vector. For example, four previous pixels are selected as context for pixels in the first row of image: $\left\{{\bar{s}}_{x,y-4,z},{\bar{s}}_{x,y-3,z},{\bar{s}}_{x,y-2,z},{\bar{s}}_{x,y-1,z}\right\}$, we repeat previous pixel values to make the size of context vector equals to four even if y<4. For pixels in the first column, the spatial context vector becomes $\left\{{\bar{s}}_{x-1,y,z},{\bar{s}}_{x-1,y+1,z},{\bar{s}}_{x-1,y+2,z},{\bar{s}}_{x-1,y+3,z}\right\}$. Similarly, context vector for pixels in the last column of the image can be written as $\left\{{\bar{s}}_{x,y-2,z},{\bar{s}}_{x,y-1,z},{\bar{s}}_{x-1,y-1,z},{\bar{s}}_{x-1,y,z}\right\}$. Besides, four pixels from previous bands co-located with the current pixel are chosen as the spectral context, denotes as $C_{l}=\left\{{\bar{s}}_{z,y,z-4},{\bar{s}}_{x,y,z-3},{\bar{s}}_{x,y,z-2},{\bar{s}}_{x,y,z-1}\right\}$. Note for prediction of the pixels in the spectral bands where z<4, we use spatial contexts only.

### 2.2. Prediction Performance Analysis

The performance of the prediction based algorithms largely depends on the choice of the context. Information theoretic analysis can provide an upper bound on the amount of compression achievable based on the specific context. The analysis employs the concept of conditional entropy, as a measure of information gain, based on a simple model of prediction process [29].

Let $X_{i}$ be a two-dimensional spectral image of the hyperspectral dataset, and $i\in \left\{1,2,\ldots ,K\right\}$, where *K* is the total number of spectral bands in the data cube. We reshape pixel value of $X_{i}$ into a vector, then the occurrences of pixels in the vector can be viewed as a random process. For a hyperspectral image having 16 bits/pixel, the first order statistical properties of $X_{i}$ is defined in terms of the probabilities $p_{j}=P\left(x=j\right),j\in \phi$, where ϕ is the set of distinct pixel values in $X_{i}$, with the range $\left[0,2^{16}-1\right]$. Then the entropy of the source can be written as [30]:

(2) $$H\left(X_{i}\right)=-\sum_{j\in \phi }p_{j}\log_{2}p_{j},$$

where $H(X_{i})$ is the minimum bit rate that lossless compression can possibly achieve using an ideal entropy coder.

The information gain of $X_{i}$ can be further reduced by exploiting the first-order statistical information of contexts. The entropy scheme $H(X_{i})$ can be easily extended to conditional entropy of band $X_{i}$ given spatial context $C_{t}$ and spectral context $C_{l}$:

(3) $$H\left(X_{i}|C_{t},C_{l}\right)=-\sum_{j\in \phi }p_{j|C_{t},C_{l}}\cdot \log_{2}\left[p_{j|C_{t},C_{l}}\right],$$

where $p_{j|C_{t},C_{l}}$ is the conditional probability $p_{j|C_{t},C_{l}}=P\left(x=j|C_{t},C_{l}\right)$. By applying the chain rule, the conditional entropy can be further rewritten as:

(4) $$H\left(X_{i}|C_{t},C_{l}\right)=H\left(X_{i},C_{t},C_{l}\right)-H\left(C_{t},C_{l}\right).$$

The conditional entropy gives the minimum achievable bit rate of $X_{i}$, given the context $C_{t}$ and $C_{l}$. In general, by exploiting the spectral and spatial correlation, we will have $H\left(X_{i}|C_{t},C_{l}\right)<H\left(X_{i}\right)$.

In practice, as stated in [14], the conditional entropy estimation becomes very inaccurate when two or more previous bands are used for prediction. It is because the conditional entropy have to estimate the joint entropy by calculating the occurrence frequencies on a very large alphabet space, i.e., ${\left(2^{16}\right)}^{N_{l}+N_{t}+1}$ in our case, where $N_{t}$ and $N_{l}$ is the number of bands used for selecting spatial and spectral context. As a consequence, a band might not contain enough pixels to provide a statistically meaningful estimation of the probabilities. [14] proposes to use the bit-planes of $X_{i}$ as a set of 16 binary sources to greatly reduce the alphabet size. However, results obtained from the binary source might not be representative of the actual alphabet sources, since the correlations between the bit-planes cannot be ignored. To solve this problem, we propose to use a neural network to extract the features from the selected contexts, which are more representative of the context sources.

## 3. Proposed Method

The proposed approach was motivated by the state of the art CCSDS-123 standard onboard compressor [12], which has been proved very efficient in lossless compression [10,12]. In CCSDS-123, the core algorithm FL is mainly a gradient-based adaptive filter. The predicted value ${\hat{s}}_{t}$ and error $\Delta_{t}$ can be expressed as:

(5) $$\begin{array}{cc} {\hat{s}}_{t} & =W_{t}^{T}U_{t},\\ \Delta_{t} & =s_{t}-{\hat{s}}_{t}, \end{array}$$

where the $W_{t}$ and $U_{t}$ are the weight vector and input context vector. Then the weight vector is updated adaptively based on $\Delta_{t}$:

(6) $$W_{t+1}=clip\left(W_{t}+\left\lfloor \frac{1}{2}\left(sgn\left[\Delta_{t}\right]\cdot 2^{-\left(\rho_{t}+\zeta \right)}\cdot {\hat{s}}_{t}+1\right)\right\rfloor ,\left\{w_{min},w_{max}\right\}\right),$$

where clip denotes the clipping of the real number to the range $\left\{w_{min},w_{max}\right\}$, and sgn is the sign function defined as $sgn=\frac{d}{dx}\left|x\right|,x\neq 0$. $\rho_{t}$ is the weight update scaling exponent, and ζ is the inter-band weight exponent offsets in the range −6≤ζ≤5.

Our goal here is to improve the traditional gradient-based adaptive filter with a neural network. The main idea is that the training a neural network can be interpreted as a nonlinear filtering procedure. Compared to the FL algorithm, the corresponding prediction value and error can be rewritten in a neural network setting:

(7) $$\begin{array}{cc} {\hat{s}}_{t} & =F_{net}\left(U_{t}\right),\\ \Delta_{t} & =F_{loss}\left(s_{t},{\hat{s}}_{t}\right), \end{array}$$

$F_{net}$ and $F_{loss}$ are the designed network and the loss function. Then weights and bias are updated by the batch gradient decent with a small learning rate:

(8) $$W_{t+1}=W_{t}-\eta \frac{\partial \Delta_{t}}{\partial W_{t}}.$$

As we can see, the prediction and the updating scheme of the neural network are very similar to the FL algorithm, which indicates that the neural network can play the role of a nonlinear adaptive filter.

With onboard data compression (with limited data for pre-training the network) in mind, we propose a filtering-based concatenated shallow network (CSNN) for predictive lossless compression. The CSNN behaves as an adaptive filter, which updates the network parameters on-the-fly with the incoming batch input. Specifically, the input samples (following a zigzag scanning order) flow through the network for just one time. The prediction error of each sample is recorded simultaneously for further mapping (to non-negative integers) and entropy coding. The weights and biases are adjusted for each batch according to the prediction errors. Algorithm 1 provides more details of the proposed adaptive scheme.
**Algorithm 1** Algorithm for filtering based CSNN adaptive prediction.1:Initialize the neural networks.2:Calculate the local sample mean using Equation (1).3:**for** t = 1:N **do** %% *N* is the number of batch, and the batch size equals to the number of columns in the spectral band.4:    Select the spatial and spectral contexts $C_{t}$ and $C_{l}$ for each pixels in batch, and prepare the data pair $\left\{\left(C_{t},C_{l}\right),s_{x,y,z}\right\}$.5:    Extract spatial and spectral features $F_{t}$ and $F_{l}$ from the contexts using one-layer shallow neural networks.6:    Concatenate the features: $F=\left[F_{t},F_{l}\right]$.7:    Predict the pixel values based on *F* using Equation (11).8:    Calculate and record the prediction error $e_{x,y,z}$ for further mapping and coding.9:    Calculate the weight updates Δw using Equation (13).10:    Adjusting the parameters in every batch: w=w+Δw.11:**end for**

We design the prediction method in such a way that the training procedure associated with most conventional neural networks based algorithms is not required. The filtering-based network aims to get the losses of each input sample rather than a well-trained network. Also, training a neural network relies on the availability of a large amount of training data, as well as an iterative optimization process of high computational complexity. In contrast, the proposed method filters each input sample only once, with a zigzag scanning order band by band. Thus, the computational time of filtering-based network is significantly lower than the conventional training-based neural network. In a nutshell, the filtering-based CSNN provides a more robust solution for a wide variety of hyperspectral datasets, without any pre-training or prior knowledge needed.

### 3.1. Concatenated Shallow Neural Network (CSNN)

**Figure 2:**
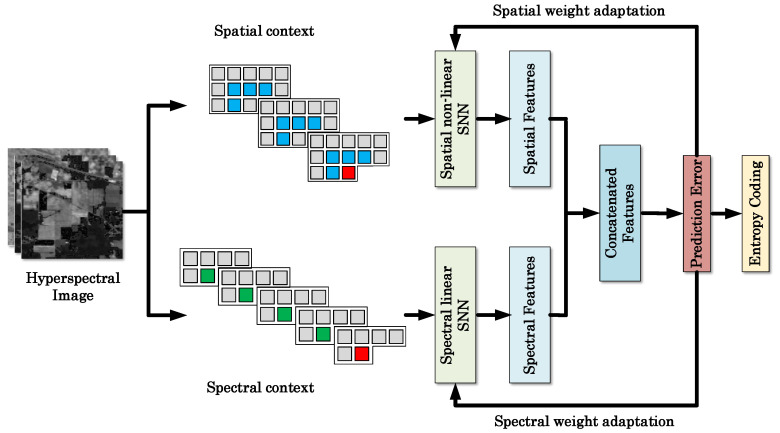
Framework of the proposed method.

Figure 2 illustrates the framework of the proposed method. The processing flow of the proposed method consists of two channels, i.e., the spatial channel and spectral channel. Both channels extract representative features from the corresponding contexts. The features from these two channels are then combined to obtain the final predicted pixel values.

The spatial contexts and spectral contexts tend to correlate with the current sample pixel to be predicted in a very different way. Conventional prediction methods either directly combine these two types of contexts, or use the spectral contexts only. In this work, we introduce two parallel shallow neural networks to learn the spatial and spectral correlations separately, in light of the good ability of neural networks for many ill-posed tasks.

**Figure 3:**
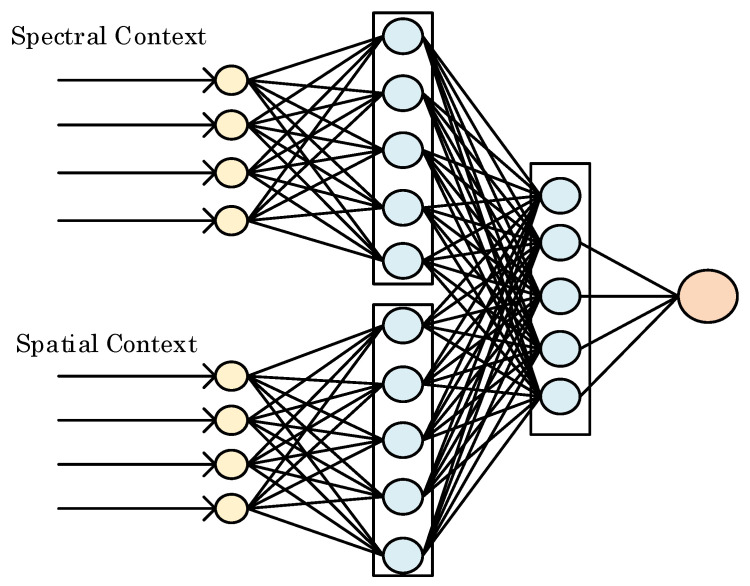
Structure of the concatenated shallow neural network.

Figure 3 shows the structure of the concatenated shallow neural network for pixel prediction. To extract spatial correlations, the hidden neurons connected with spatial context converts the input into a series of feature maps via a non-linear process,

(9) $$F_{t}=ReLU\left(w_{t}\cdot C_{t}+b_{t}\right),$$

where $F_{t}$ is the extracted spatial features, and $w_{t}$ and $b_{t}$ denote the corresponding weight and bias. A Rectified Linear Unit, $ReLU=\max\left(0,x\right)$, is used as the activation function for spatial channel. In this work, a 3 × 4 spatial context matrix is converted to a 1 × 5 feature vector by means of five connected neurons.

For the spectral channel, exactly the same number of hidden neurons are used for extracting the spectral feature, however, the *ReLU* activation function is not used here to obtain the spectral feature $F_{l}$ below, based on our observation in extensive empirical study that the spectral contexts tend to be correlated in a less non-linear fashion than the spatial contexts.

(10) $$F_{l}=w_{l}\cdot C_{l}+b_{l}.$$

Similarly, $w_{l}$ and $b_{l}$ in the equation above denote the corresponding weights and biases.

Note that the neural networks are employed for both channels as opposed to deep neural networks. Although deep networks are capable of capturing high dimensional features, they might suffer from the overfitting problem. To predict a spectral band whose context changes rapidly, optimization of the deep network might be trapped by local optima and thus fail to react promptly. Besides, since a large number of the weights and biases need be adjusted by prediction errors using back propagation, training of deep network may be time consuming.

**Figure 4:**
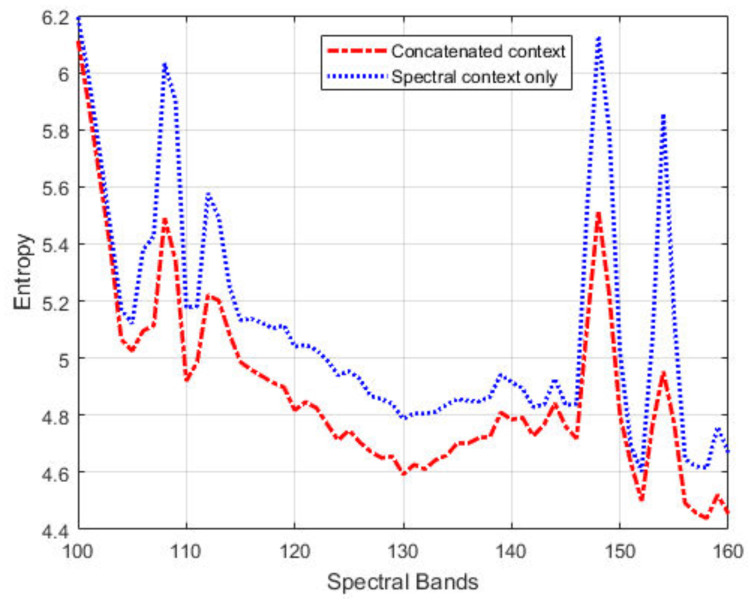
Entropy of the prediction errors across spectral bands 100 to 160 of the IP dataset, using the proposed concatenated contexts (in red) and the spectral contexts only (in blue) for prediction.

The extracted features from two channels are concatenated together denote as $F=\left[F_{t},F_{l}\right]$. The combined features jointly decide the final predicted pixel value with a linear output layer,

(11) $$\hat{s}=w_{f}\cdot F+b_{f}$$

where $w_{f}$ and $b_{f}$ are the weights and biases for the final layer. Thus, the CSNN model contains two hidden layers: the first hidden layer extracts spatial and spectral features, and the second concatenated hidden layer for final pixel value prediction. Figure 4 shows the first-order entropies of the prediction residuals for a segment of the IP dataset, with the joint spatial/spectral contexts and spectral contexts only, respectively. We can see that combined contexts allow for more accurate prediction (i.e., low entropy values). The improvement in prediction is especially pronounced for band images with large pixel-intensity variations, for example, entropy reduction by 0.6 bit for band 148 and 0.9 bit for band 154.

The CSNN is a typical end-to-end fully connected neural network, with the weights and biases being updated by the Adadelta optimizer [31] with the $L_{1}$ loss function:

(12) $$L_{F}=\frac{1}{N}\sum_{n=1}^{N}\left|s_{x,y,z}-{\hat{s}}_{x,y,z}\right|.$$

Note that the $L_{1}$ loss function was adopted since our study found that it can lead to lower residual entropies than the $L_{2}$ loss function, which favors quality assessment based on the mean square errors. If we set $g_{t}=\frac{\partial L_{F}\left(t\right)}{\partial w_{t}}$ to be the gradient of the parameters at *t*-th input data, the update $\Delta w_{t}$ can be calculated as follows:

(13) $$\Delta w_{t}=-\frac{RMS[\Delta w_{t-1}]}{RMS[g_{t}]}\cdot g_{t},$$

where RMS is the root mean square, which is defined as $RMS\left[g_{t}\right]=\sqrt{E[g_{t}^{2}]+\epsilon }$. $E[g_{t}^{2}]$ is an exponentially decaying average of the squared gradients:

(14) $$E\left[g_{t}^{2}\right]=\rho E\left[g_{t-1}^{2}\right]+\left(1-\rho \right)g_{t}^{2},$$

where ρ is a decay constant and ϵ is added to the numerator of RMS to ensure progress continues to be made even if the previous updates become small. We set ρ=0.95 and $\epsilon =e^{-6}$ in the simulations. The code of CSNN model can be found at [32].

### 3.2. Entropy Coding

After prediction, all the residuals are mapped to non-negative values [33] and then coded into bitstream losslessly using a Golomb Rice Codec (GRC) [34]:

(15) $$f\left(n\right)=\left\{\begin{array}{cc} 2n, & n\geq 0\\ -2n-1, & n<0 \end{array}\right.$$

where *n* refers to the value of the prediction residual. Note GRC is selected as the entropy coder due to its computational efficiency. We observed that arithmetic coding [35] can offer slightly lower bitrates, albeit at a much higher computational cost.

Besides the GR codewords, there is other side information that needs to be transferred to the decoder in order to recover the original data losslessly. For example, the weights and biases that initialize the neural networks need to be encoded too. Since the CSNN model has less than 20 neurons, such side information becomes negligible and thus it is not included in the total bit rates reported in the following.

## 4. Simulations Results

We tested the proposed method on five public hyperspectral datasets [36] and the standard CCSDS test datasets. We selected 20 datasets from the CCSDS test sets with different collecting instruments: Atmospheric Infrared Sounder (AIRS), Airborne Visible/Infrared Imaging Spectrometer (AVIRIS), SWIR Full Spectrum Imager (SFSI), and Compact Airborne Spectrographic Imager (CASI). The test results are compared with the state-of-the-art CCSDS-123 method. Before presenting the results, we first provide a convergence analysis on the proposed model, and discuss the sensitivity of parameter initialization on the compression result.

### 4.1. Convergence Analysis and Parameter Sensitivity

Deep neural network is the mainstream technique for many machine learning tasks. Despite its success, theoretically explaining why deep neural networks can be efficiently trained in practice using simple gradient-based methods is still an open problem. Filtering the input data by the CSNN model is similar to the training process, which requires optimizing a complex non-convex problem. Over the past few years, much research has been devoted to this problem [37,38,39,40,41]. Based on these existing work, we found that the convergence of the neural network is closely related to the choice of hyperparameters such as the number of hidden layers, the number of hidden units and the learning rate. Although training a network seems intractable, [37] provides some tricks to determine the hyper-parameters in the model.

**Figure 5:**
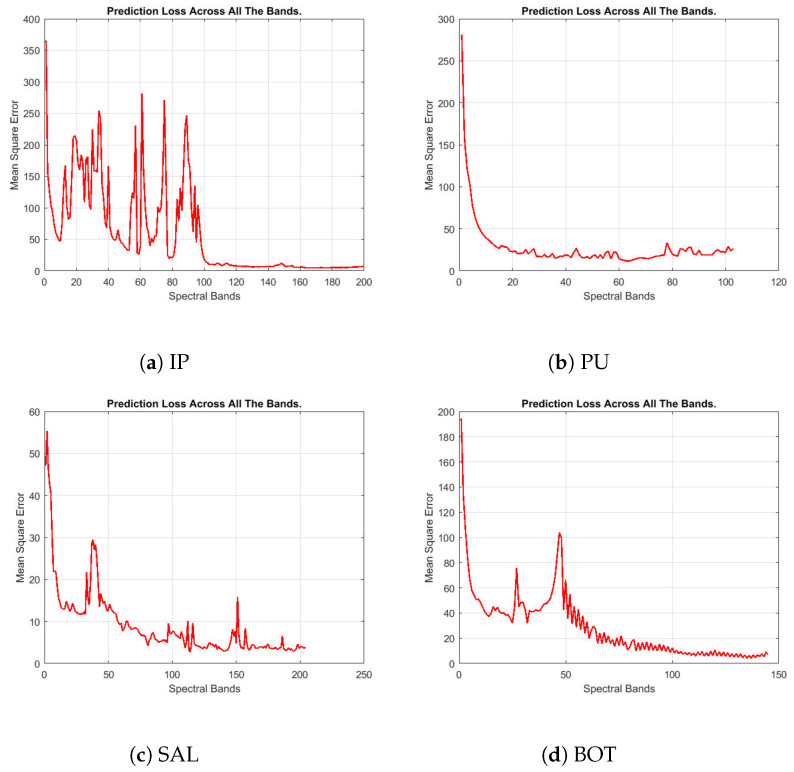
Variation of mean square errors across all the spectral bands on four hyperspectral datasets using filtering-based CSNN.

The convergence of neural networks is studied in [39,40]. An early convergence of filtering-based network can provide much smaller prediction loss, and lower compression bitrates as a return. To demonstrate the convergence of filtering-based CSNN model, we perform the filtering-based CSNN on four public hyperspectral datasets: *Indian Pines* (IP), *Pavia University* (PU), *Salinas* (SAL), and *Botswana* (BOT). Figure 5 shows the prediction loss in Mean Square Error (MSE) across all the spectral bands.

Clearly, the prediction loss curves of four datasets show convergence to relatively small error values. For example, the losses of the PU dataset decrease to a very low level after filtering the first 20 bands. For other datasets, even if the losses fluctuate intensely at the beginning like IP dataset, the losses converged to small values after half of the data have been filtered. This demonstrates the ability of the proposed CSNN model to find a fairly good solution with only a single iteration on the data. We believe that the similarity (correlation) of data samples among different spectral bands helps accelerate the convergence of the model.

**Figure 6:**
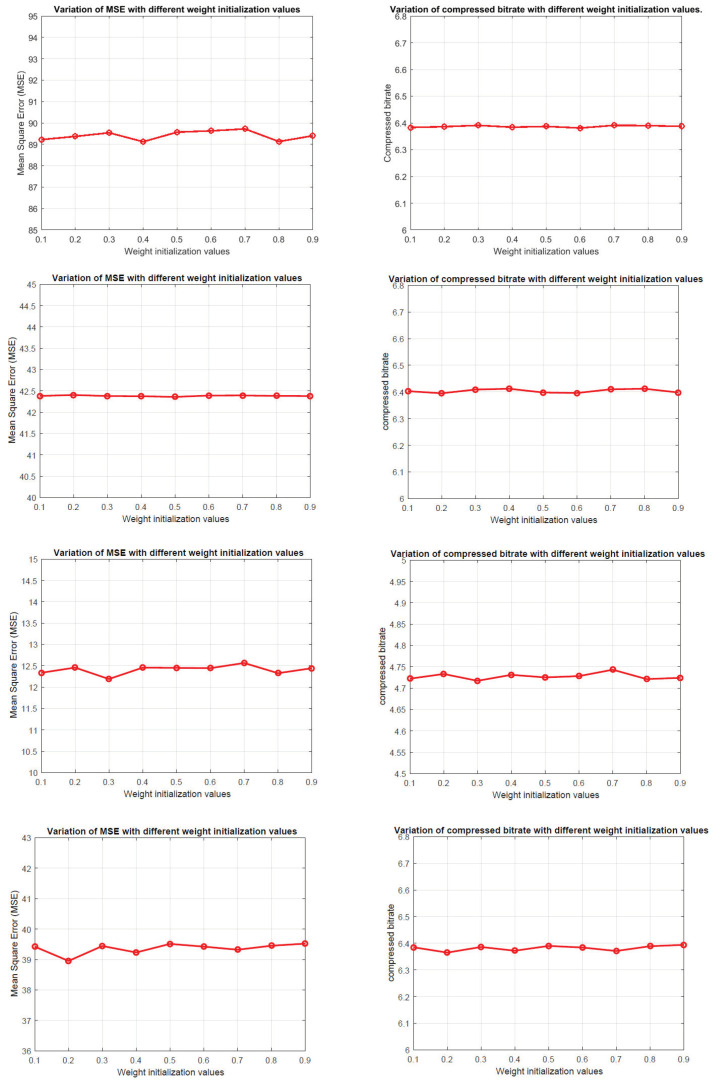
Variations of the mean square errors (left side) and compressed bitrate (right side) with different weight initialization values on the four datasets using filtering-based CSNN. Two figures in the first row is the variation of IP dataset, the remaining rows are PU, SAL and BOT, respectively.

Another factor that can influence the convergence is the initialization of parameters in the network. In our simulations, we select *Xavier* initialization [42], which is one of the most commonly used initialization methods for the neural network. In *Xavier*, all the weights initialize independently from a zero-mean, unit variance distribution. It is interesting to see how different weight initialization methods would affect prediction loss and entropy. Thus, we conduct several experiments by assigning network with weight values ranging from 0.1 to 0.9. Figure 6 shows the variations of MSE values and compression bitrates on four different datasets using proposed method. Note the weight parameters include the weights and biases in each layer.

We can see that the MSE values and compressed bitrates do not appear to be very sensitive to change of weight parameters. For example, the MSE values of IP dataset range from 89 to 90 with different initialized weight values. For other datasets, the value of MSE and bitrate all fluctuated within a small range. The testing results are consistent with the conclusion in [40], where the gradient-based linear neural network has a strong convergence ability. It also indicates the filtering-based CSNN is robust to the initial condition.

### 4.2. Simulations on Five Public Hyperspectral Datasets

The five hyperspectral image datasets include IP, PU, SAL, BOT and *Kennedy Space Center* (KSC). All these datasets contain 12-bits non-calibrated raw images. More detailed information of these data sets are given in Table 1.

We select three representative adaptive filtering methods to benchmark the proposed method:

- The conventional Least Mean Square (LMS) filter, widely used for lossless compression of hyperspectral data due to its simplicity.
- The MCC-LMS filter proposed in [15], which replaces the LMS cost function with the correntropy function, and achieves significant compression on regions of interest in hyperspectral images.
- The state-of-the-art Fast Lossless (FL) method, firstly proposed in [10], and then adopted by the new CCSDS standard for lossless hyerpsectral data compression.

As we can see in Table 2, the proposed CSNN method achieves the lowest bitrates on all five hyperspectral image datasets, where the LMS method has the highest bitrates. Specifically, the CSNN method improves by nearly 0.2 bit/pixel and 0.25 bit/pixel on average on the FL and MCC-LMS methods, respectively, with a more significant reduction of 0.58 bit/pixel over the LMS method. The CSNN seems to provide more efficient compression by exploiting jointly the spatial and spectral correlations from the contexts.

**Figure 7:**
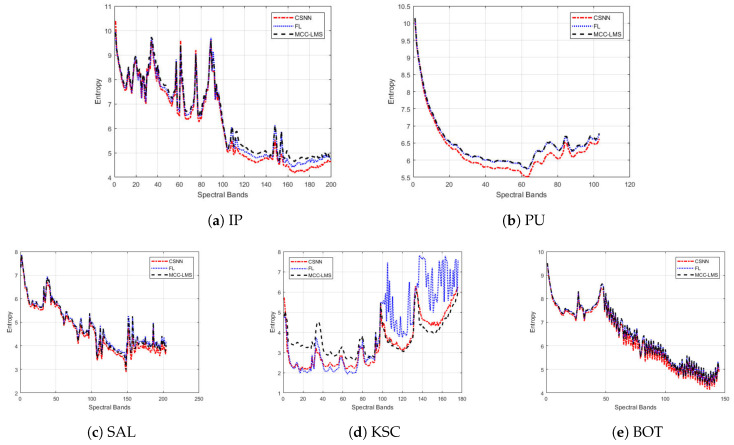
Entropy of the prediction residuals across all the spectral bands of five hyperspectral datasets.

In terms of the prediction residuals, Figure 7 shows that the CSNN method consistently achieves the lowest entropies on the most of bands of IP, PU, SAL and BOT datasets, with more obvious improvement for the last 50 bands. For example, in Figure 7b, the curves of the last 50 bands of MCC-LMS and FL methods almost overlap, while the CSNN curve goes much lower. The curves for the KSC dataset in Figure 7d exhibit significant fluctuations for all three methods. This data set contains a substantial amount of impulse noise, which might cause many sudden changes of the contexts. For example, the residual entropy fluctuates rapidly after band 100. But still, the proposed method seems to be the most stable one among the four methods. By considering also the compression bitrates of the KSC dataset in Table 2, we can see that the adaptive filtering methods (including the proposed method) are not robust enough to data with noise.

### 4.3. Simulations on CCSDS Test Datasets

**Figure 8:**
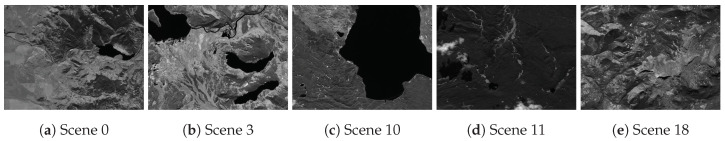
Some scenes in the 16-bit uncalibrated “Yellow Stone” hyperspectral images of the AVIRIS 2006 dataset.

The CCSDS hyperspectral and multispectral test corpus [43] has been publicly available for hyperspectral images compression testing and evaluation. The corpus includes images from many different instruments. To diversify the testing datasets, we selected 20 hyperspectral datasets from instruments AVIRIS, AIRS, SFSI and CASI for further evaluation of the algorithms. Seven hyperspectral images are from AVIRIS instrument, which includes five 16-bit non-calibrated Yellowstone scenes and two 12-bit scenes. The AIRS instrument has ten scenes, each scene has 1501 spectral bands and 90 lines with a width of 153 pixels. The remaining three images are from instruments SFSI and CASI. Table 3 provides detailed information about the selected datasets. As an example, The grayscale versions of the five AVIRIS Yellowstone scenes are shown in Figure 8.

Lossless hyperspectral image compression techniques can be separated into two main categories: transform-based compression and prediction-based compression. Transform-based techniques achieve compression by taking advantage of frequency domain representation of images (e.g., based on wavelet transforms). On the other hand, predictive compression performs directly on pixel domain, followed by entropy coding on the prediction residuals (e.g., by using the Golomb-Rice codes). We selected a total of seven lossless compressors from both categories: JPEG2000 [3], JPEG-LS [2], LUT [6], European Space Agency (ESA) [44], CCSDS-122 [45], MCC-LMS [15], CCSDS-123 [12]. Note the state-of-the-art predictive compressor CCSDS-123 is also provided for comparison.

Table 4 provides the lossless coding results for all the images in terms of the bit rate (in bpppc). The compression efficiency of each algorithm can be appreciated by observing the degree to which its resulting bit rate falls below the bit depth of the original images. We can see that the overall performance of the proposed filtering-based CSNN method exceeds other state-of-the-art methods included in the comparison.

For 16 bpppc non-calibrated AVIRIS Yellowstone scenes, the filtering-based CSNN model outperforms CCSDS-123 and MCC-LMS by 0.12 bpppc and 0.17 bpppc on average, respectively. When compared with the transform-based compression, the coding gain of CSNN is larger than JPEG2000 and CCSDS-122 by 0.52 bpppc and 0.60 bpppc, respectively.

For 12 bpppc non-calibrated AVIRIS scene (Hawaii and Maine), the coding performance of proposed model is comparable with CCSDS-123. Specifically, the compressed bitrate of CSNN is 0.03 bpppc higher than CCSDS-123, but much lower than other methods. Compared with other 12 bpppc images with different instruments, the Hawaii and Maine scenes have relatively smaller pixel values. For example, the average pixel value of Hawaii and Maine scene is 267.10 and 328.75, respectively. But for AIRS-gran9 image, the average pixel value is 2091. This indicates that the filtering-based CSNN would obtain more compression gain for images with higher pixel values. These results are also consistent with the property of the gradient-based network, where the neural network can make a quick response for large variations in the data. However, linear predictors, such as CCSDS-123, might be more suitable for slowly changing data with small values.

For other images from AIRS, SFSI and CASI instruments, the filtering-based CSNN model also provides superior performance compared to other prominent predictive coding techniques. To summarize, the results show that the proposed filtering-based CSNN yields the best overall achievement as compared to other predictive-based compressors. It offers additional desirable features such as no pre-training involved in compression procedure, thereby making it an appealing approach for lossless hyperspectral image compression.

**Figure 9:**
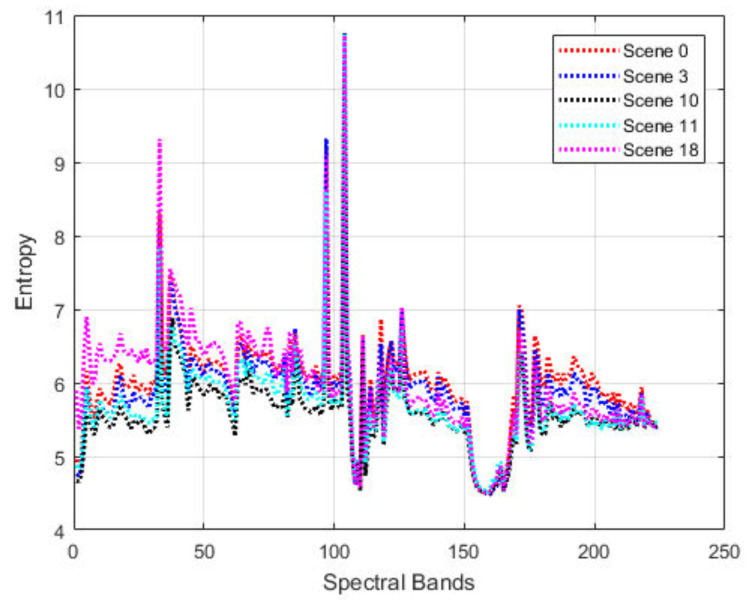
Entropies of prediction residuals of the proposed method on 16-bit uncalibrated “Yellow Stone” hyperspectral datasets.

Figure 9 shows the residual entropy variations of the “Yellow Stone” scenes. It is interesting to observe that five distinct scenes seem to follow a similar trend spectral band around 160 are almost the same. This indicates the robust prediction performance that can be achieved by jointly considering both the spatial and spectral contexts using the proposed method. There is the potential to use transfer learning to exploit such a similarity to further improve the compression performance.

### 4.4. Computational Complexity

The computation of the proposed CSNN method includes feed-forward propagation and back propagation. Note the filtering of CSNN model is mainly implemented with multiplication of matrices. Assume there are *i* nodes in the input layer, corresponding to *i* context pixels being fed to the network, and *j* and *k* denotes the number of nodes in the two hidden layers, and *l* denotes the number of nodes in the output layer. In a four-layer neural network, there are three weight matrices to represent weights between these layers. For example, $W_{ji}$ is a weight matrix with *j* rows and *i* columns, which contains the weights going from layer *i* to layer *j*. In a feed forward pass, propagating a sample from layer *i* to layer *j* takes O(j·i) time complexity, thus the overall time complexity from the input layer to the output layer becomes O(j·i+k·j+l·k). The back propagation starts from the last layer of the model. Similar to the feed forward pass, the time complexity of the back propagation is given by O(l·k+k·j+j·i). We can see that the computational complexity of the neural network largely depends on the number of hidden layers and the number of nodes in each layer. Also, as shown in Equations (9) and (10), activation function is only needed for the spatial channel, with very light computation required for the ReLU function. The computation time for compressing the IP dataset takes 0.8 seconds/band, the experiments were carried out on a Thinkpad laptop with Intel Core i5 CPU and 8GB installed memory, running Windows 7 Professional (64-bit operating system). Note the matrix operations can be greatly parallelized by GPUs to further reduce the computation time.

## 5. Conclusions

Hyperspectral imaging has found increasingly widespread applications in a growing number of fields. Data compression especially the lossless compression plays a key role in efficiently transmitting and storing the hyperspectral data.

In this paper, we design a shallow neural network to extract and combine spatial and spectral contexts to improve predictive coding. The filtering-based neural network requires no training and has low computational complexity. Extensive simulation results on public hyperspectral images and the standard CCSDS calibrated and uncalibrated test datasets demonstrate that the proposed method provides higher compression than several other state-of-the-art methods.

The proposed method provides a tradeoff between computational complexity and coding performance that makes it an appealing approach for hyperspectral lossless data compression. Further research will study how to take advantage of the similarity between different scenes to further improve the compression efficiency.

## Figures and Tables

**Table 1:** Detailed information of public hyperspectral image data sets.

Dataset	Size	Bit-per-Pixel	Sensor
IP	145×145×200	16	AVIRIS
PU	610×340×103	16	ROSIS
SAL	512×217×204	16	AVIRIS
KSC	512×614×176	16	AVIRIS
BOT	1476×256×145	16	AVIRIS

**Table 2:** Lossless compression bitrates (in bits/pixel) on five hyperpsectral data sets.

Dataset	LMS	MCC-LMS	FL	CSNN
IP	6.69	6.64	6.58	**6.38**
PU	6.66	6.58	6.55	**6.40**
SAL	4.94	4.92	4.87	**4.73**
KSC	6.31	4.82	4.74	**4.36**
BOT	6.58	6.54	6.50	**6.38**
Average	6.23	5.90	5.85	**5.65**

**Table 3:** Dataset information for AVIRIS, AIRS, SFSI and CASI sensors. *z* is the number of spectral bands, *y* is the height, *x* is the Width. bpppc is bits per pixel per component, and “raw” is image type.

Instrument	Images
AVIRIS (16 bpppc, raw)	Yellowstone Scene: 0, 3, 10, 11,18
z=224, y=512	Hawaii (x=614, 12bpppc)
x=680	Maine (12 bpppc)
	AIRS-gran9
	AIRS-gran16
	AIRS-gran60
AIRS (12-14 bpppc, raw)	AIRS-gran82
z=1501	AIRS-gran120
y=90	AIRS-gran126
x=153	AIRS-gran129
	AIRS-gran151
	AIRS-gran182
	AIRS-gran193
SFSI (12 bpppc, raw)	Mantar scene
z=240,y=496,x=140	
CASI (12 bpppc, raw)	CASI-t0477f06 (y=406,x=1225)
z=72	CASI-t0180f07 (y=405,x=2852)

**Table 4:** Lossless compression bitrates (in bits/pixel) for JPEG2000, JPEG-LS, LUT, ESA, CCSDS-122, MCC-LMS, CCSDS-123 and filtering-based CSNN on CCSDS test datasets.

Data Set	JPEG2000	JPEG-LS	LUT	ESA	CCSDS-122	MCC-LMS	CCSDS-123	CSNN
AVIRIS 16 bpppc								
AVIRIS-YS 0	6.65	6.95	7.15	6.45	6.70	6.25	6.19	**6.05**
AVIRIS-YS 3	6.47	6.83	6.93	6.30	6.54	6.11	6.06	**5.92**
AVIRIS-YS 10	5.80	6.16	6.23	5.62	5.93	5.62	5.58	**5.58**
AVIRIS-YS 11	6.27	6.48	6.80	6.03	6.36	5.88	5.83	**5.75**
AVIRIS-YS 18	6.71	6.94	7.22	6.39	6.76	6.31	6.21	**6.01**
Average	6.38	6.67	6.87	6.16	6.46	6.03	5.98	**5.86**
AVIRIS 12 bpppc								
Hawaii	2.99	3.27	3.38	2.94	3.29	3.15	**2.62**	2.67
Maine	3.09	3.36	3.51	3.07	3.36	3.16	**2.73**	2.75
Average	3.04	3.32	3.44	3.01	3.33	3.15	**2.68**	2.71
AIRS								
AIRS-gran9	4.58	4.47	5.53	4.59	4.94	4.67	4.22	**4.09**
AIRS-gran16	4.52	4.68	5.41	4.53	4.90	4.63	4.20	**4.04**
AIRS-gran60	4.80	4.98	5.85	4.81	5.16	4.80	4.38	**4.27**
AIRS-gran82	4.40	4.56	5.21	4.38	4.79	4.72	4.13	**3.97**
AIRS-gran120	4.65	4.80	5.49	4.66	5.01	4.64	4.29	**4.17**
AIRS-gran126	4.81	4.99	5.82	4.79	5.16	4.82	4.40	**4.28**
AIRS-gran129	4.43	4.53	5.32	4.45	4.80	4.53	4.14	**3.96**
AIRS-gran151	4.80	4.95	5.90	4.81	5.14	4.80	4.41	**4.32**
AIRS-gran182	4.85	4.96	6.26	4.90	5.21	4.83	4.43	**4.29**
AIRS-gran193	4.83	4.99	5.75	4.84	5.17	4.72	4.42	**4.29**
Average	4.67	4.82	5.66	4.68	5.03	4.71	4.30	**4.17**
CASI								
casi-t0180f07	5.16	5.23	5.49	4.96	5.30	4.84	4.86	**4.68**
casi-t0477f06	5.57	5.44	5.82	5.23	5.61	5.08	5.18	**4.90**
Average	5.37	5.33	5.65	5.10	5.46	4.96	5.02	**4.79**
SFSI								
Mantar	4.56	5.02	5.43	4.91	4.90	4.81	4.67	**4.54**

## References

[1] Roger R.E., Cavenor M. (1996). Lossless compression of AVIRIS images. IEEE Trans. Image Process..

[2] Weinberger M.J., Seroussi G., Sapiro G. (2010). The LOCO-I lossless image compression algorithm: Principles and standardization into JPEG-LS. IEEE Trans. Image Process..

[3] Gonzalez-Conejero J., Bartrina-Rapesta J., Serra-Sagrista J. (2010). JPEG 2000 encoding of remote sensing multispectral images with no-data regions. IEEE Geosci. Remote Sens. Lett..

[4] Wu X., Memon N. (2000). Context-based lossless interband compression - extending CALIC. IEEE Trans. Image Process..

[5] Magli E., Olmo G., Quacchio E. (2004). Optimized onboard lossless and near-lossless compression of hyperspectral data using CALIC. IEEE Trans. Geosci. Remote Sens..

[6] Mielikainen J. (2006). Lossless compression of hyperspectral images using lookup tables. IEEE Signal Process. Lett..

[7] Mielikainen J., Toivanen P. (2008). Lossless compression of hyperspectral images using a quantized index to lookup tables. IEEE Geosci. Remote Sens. Lett..

[8] Amrani N., Sagrista J.S., Laparra V., Marcellin M.W., Malo J. (2016). Regression Wavelet Analysis for Lossless Coding of Remote-Sensing Data. IEEE Trans. Geosci. Remote Sens..

[9] Cortes S.A., Rapesta J.B., Sagrista J.S. (2018). Multilevel Split Regression Wavelet Analysis for Lossless Compression of Remote Sensing Data. IEEE Geosci. Remote Sens. Lett..

[10] Klimesh M. (2005). Low-Complexity Lossless Compression of Hyperspectral Imagery via Adaptive Filtering.

[11] Rizzo F., Carpentieri B., Motta G., Storer J.A. (2005). Low-Complexity lossless compression of hyperspectral imagery via linear prediction. IEEE Signal Process. Lett..

[12] CCSDS Low-Complexity Lossless and Near-Lossless Multispectral and Hyperspectral Image Compression CCSDS 123.0-B-2, ser. Blue Book, February 2019. https://public.ccsds.org/Pubs/123x0b2c1.pdf.

[13] Lin C.C., Hwang Y.T. (2010). An efficient lossless compression scheme for hyperspectral images using two-stage prediction. IEEE Geosci. Remote Sens. Lett..

[14] Magli E. (2009). Multiband lossless compression of hyperspectral images. IEEE Trans. Geosci. Remote Sens..

[15] Shen H., Pan W.D. Predictive lossless compression of regions of interest in hyperspectral image via maximum correntropy criterion based least mean square learning. Proceedings of the 2016 IEEE International Conference on Image Processing (ICIP).

[16] Wang H., Babacan S.D., Sayood K. (2007). Lossless hyperspectral-image compression using context-based conditional average. IEEE Trans. Geosci. Remote Sens..

[17] Kiely A.B., Klimesh M.A. (2009). Exploiting calibration-induced artifacts in lossless compression of hyperspectral imagery. IEEE Trans. Geosci. Remote Sens..

[18] Mielikainen J., Toivanen P. (2003). Clustered DPCM for the lossless compression of hyperspectral images. IEEE Trans. Geosci. Remote Sens..

[19] Mielikainen J., Huang B. (2012). Lossless compression of hyperspectral images using clustered linear prediction with adaptive prediction length. IEEE Geosci. Remote Sens. Lett..

[20] Aiazzi B., Alparone L., Baronti S., Lastri C. (2007). Crisp and fuzzy adaptive spectral prediction for lossless and near-lossless compression of hyperspectral imagery. IEEE Geosci. Remote Sens. Lett..

[21] Wang L., Zhang T., Fu Y., Huang H. (2019). HyperReconNet: Joint Coded Aperture Optimization and Image Reconstruction for Compressive Hyperspectral Imaging. IEEE Trans. Geosci. Remote Sens..

[22] Choi I., Jeon D.S., Nam G., Gutierrez D., Kim M.H. (2017). High-quality hyperspectral reconstruction using a spectral prior. ACM Trans. Graph..

[23] Signoroni A., Savardi M., Baronio A., Benini S. (2019). Deep Learning Meets Hyperspectral Image Analysis: A Multidisciplinary Review. J. Imaging.

[24] Haut J.M., Gallardo J.A., Paoletti M.E., Cavallaro G., Plaza J., Plaza A., Riedel M. (2019). Cloud Deep Networks for Hyperspectral Image Analysis. IEEE Trans. Geosci. Remote Sens..

[25] Valsesia D., Magli E. (2019). High-Throughput Onboard Hyperspectral Image Compression With Ground-Based CNN Reconstruction. IEEE Trans. Geosci. Remote Sens..

[26] Kumar S., Chaudhuri S., Banerjee B., Ali F. Onboard hyperspectral image compression using compressed sensing and deep learning. Proceedings of the 2018 IEEE European Conference on Computer Vision (ECCV).

[27] Dusselaar R., Paul M. (2012). A block-based inter-band predictor using multilayer propagation neural network for hyperspectral image compression. arXiv.

[28] Jiang Z., Pan W.D., Shen H. LSTM Based Adaptive Filtering for Reduced Prediction Errors of Hyperspectral Images. Proceedings of the 2018 6th IEEE International Conference on Wireless for Space and Extreme Environments (WiSEE 2018).

[29] Shen H., Jiang Z., Pan W.D. (2018). Efficient Lossless Compression of Multitemporal Hyperspectral Image Data. J. Imaging.

[30] Cover T.M., Thomas J.A. (2006). Elements of Information Theory.

[31] Zeiler M.D. (2012). Adadelta: An adaptive learning rate method. arXiv.

[32] CSNN The Code of CSNN Structure. https://github.com/jj574435561/imagecomCSNN.

[33] CCSDS Lossless Multispectral & Hyperspectral Image Compression CCSDS 123.0-B-1, ser. Blue Book, May 2012. https://public.ccsds.org/Pubs/123x0b1ec1.pdf.

[34] Rice R.F. (1979). Some Practical Universal Noiseless Coding Techniques.

[35] Witten I.H., Neal R.M., Cleary J.G. (1987). Arithmetic coding for data compression. Commun. ACM.

[36] GIC Hyperspectral Remote Sensing Scenes Data. http://www.ehu.es/ccwintco/index.php?title=HyperspectralRemoteSensingScenes.

[37] LeCun Y., Bottou L., Orr G.B., Muller K. (1998). Efficient BackProp. Neural Networks: Tricks of the Trade (Outgrowth of a 1996 NIPS Workshop).

[38] Soudry D., Carmon Y. (2016). No bad local minima: Data independent training error guarantees for multilayer neural networks. arXiv.

[39] Li Y., Yuan Y. (2017). Convergence Analysis of Two-layer Neural Networks with ReLU Activation. arXiv.

[40] Arora S., Cohen N., Golowich N., Hu W. (2018). A Convergence Analysis of Gradient Descent for Deep Linear Neural Networks. arXiv.

[41] Shamir O. (2019). Exponential Convergence Time of Gradient Descent for One-Dimensional Deep Linear Neural Networks. arXiv.

[42] Glorot X., Bengio Y. Understanding the difficulty of training deep feedforward neural networks. Proceedings of the Thirteenth International Conference on Artificial Intelligence and Statistics (AISTAT).

[43] The Consultative Committee for Space Data Systems Hyperspectral and Multispectral Test Images. http://cwe.ccsds.org/sls/docs/sls-dc/123.0-B-Info/TestData.

[44] Abrardo A., Barni M., Bertoli A., Grimoldi R. (2011). Low-Complexity Approaches for Lossless and Near-Lossless Hyperspectral Image Compression. Satellite Data Compression.

[45] CCSDS Image Data Compression CCSDS 122.0-B-2, ser. Blue Book, September 2017. https://public.ccsds.org/Pubs/122x0b2.pdf.

